# Binding of NIR-conPK and NIR-6T to Astrocytomas and Microglial Cells: Evidence for a Protein Related to TSPO

**DOI:** 10.1371/journal.pone.0008271

**Published:** 2009-12-18

**Authors:** Michelle Sexton, Grace Woodruff, Eiron Cudaback, Faith R. Kreitzer, Cong Xu, Yi Hsing Lin, Thomas Möller, Mingfeng Bai, H. Charles Manning, Darryl Bornhop, Nephi Stella

**Affiliations:** 1 Bastyr University, Seattle, Washington, United States of America; 2 Department of Pharmacology, University of Washington, Seattle, Washington, United States of America; 3 Neurobiology Undergraduate Program, University of Washington, Seattle, Washington, United States of America; 4 Department of Neurology, University of Washington, Seattle, Washington, United States of America; 5 Mallinckrodt Institute of Radiology School of Medicine, Washington University, St. Louis, Missouri, United States of America; 6 Vanderbilt University Institute of Imaging Science (VUIIS), Vanderbilt University Medical Center, Nashville, Tennessee, United States of America; 7 Department of Radiology and Radiological Sciences, Department of Biomedical Engineering, and Program in Chemical and Physical Biology, Vanderbilt University Medical Center, Nashville, Tennessee, United States of America; 8 Department of Chemistry, Vanderbilt University, Nashville, Tennessee, United States of America; 9 Psychiatry and Behavioral Sciences, University of Washington, Seattle, Washington, United States of America; University of Maryland School of Pharmacy, United States of America

## Abstract

PK 11195 and DAA1106 bind with high-affinity to the translocator protein (TSPO, formerly known as the peripheral benzodiazepine receptor). TSPO expression in glial cells increases in response to cytokines and pathological stimuli. Accordingly, [^11^C]-PK 11195 and [^11^C]-DAA1106 are recognized molecular imaging (MI) agents capable of monitoring changes in TSPO expression occurring *in vivo* and in response to various neuropathologies.

Here we tested the pharmacological characteristics and TSPO-monitoring potential of two novel MI agents: NIR-conPK and NIR-6T. NIR-conPK is an analogue of PK 11195 conjugated to the near-infrared (NIR) emitting fluorophore: IRDye 800CW. NIR-6T is a DAA1106 analogue also conjugated to IRDye 800CW.

We found that NIR-6T competed for [^3^H]-PK 11195 binding in astrocytoma cell homogenates with nanomolar affinity, but did not exhibit specific binding in intact astrocytoma cells in culture, indicating that NIR-6T is unlikely to constitute a useful MI agent for monitoring TSPO expression in intact cells. Conversely, we found that NIR-conPK did not compete for [^3^H]-PK 11195 binding in astrocytoma cell homogenate, but exhibited specific binding in intact astrocytoma cells in culture with nanomolar affinity, suggesting that NIR-conPK binds to a protein distinct, but related to, TSPO. Accordingly, treating intact astrocytoma cells and microglia in culture with cytokines led to significant changes in the amount of NIR-conPK specific binding without corresponding change in TSPO expression. Remarkably, the cytokine-induced changes in the protein targeted by NIR-conPK in intact microglia were selective, since IFN-γ (but not TNFα and TGFβ) increased the amount of NIR-conPK specific binding in these cells.

Together these results suggest that NIR-conPK binds to a protein that is related to TSPO, and expressed by astrocytomas and microglia. Our results also suggest that the expression of this protein is increased by specific cytokines, and thus allows for the monitoring of a particular subtype of microglia activation.

## Introduction

Molecular imaging (MI) agents allow for the non-invasive monitoring of molecular events in intact cells and tissues. They include high affinity receptor ligands that are labeled with radioactive isotopes or conjugated to biocompatible imaging moieties. Thus, the signal emitted by MI agents – be it radioactivity or light–fluctuates in parallel with any change in the expression of the targeted receptor.

MI agents targeting the mitochondrial translocator protein (TSPO, formerly known as the peripheral benzodiazepine receptor [1]) have been developed and the signal that they emit shown to fluctuate in parallel with changes in TSPO expression in glial cells, as well as the progression of some neuropathologies. For example, [^11^C]-PK 11195 reliably monitors increases in TSPO expression in brain tumors (including malignant astrocytomas), as well as in the activated microglia found in patients with multiple sclerosis, stroke, epilepsy, Alzheimer's disease, Huntington's disease and AIDS [2], [3], [4], [5], [6], [7], [8], [9], [10]. Another MI agent, [^11^C]-DAA1106, which exhibits even higher affinity for TSPO than PK 11195, is also used to monitor TSPO expression in activated microglia [11], [12], [13], [14], [15].

Evidence suggests that moieties emitting light in the near infrared range (NIR, 700–1000 nm) hold promise for the development of MI agents, and in fact several such MI agents have been tested for their ability to monitor molecular events both *in vitro* and *in vivo* [16], [17], [18]. NIR fluorescence can be detected with high sensitivity and is relatively transparent to biological matrices (lipids, water and hemoglobin neither absorb nor auto-fluoresce significantly in the NIR range [19]). However, it is important to note that moieties emitting NIR fluorescence are hydrophobic and large in mass, and therefore their conjugation to receptor ligands may affect the ligand's affinity and selectivity towards its target. Thus the pharmacological characteristics of any newly developed MI agent emitting NIR fluorescence – their purity and especially their affinity and selectivity at the targeted receptor – should be systematically verified.

The present study focuses on two new MI agents originally designed to target TSPO: chemical analogues of PK 11195 and DAA1106 conjugated to a NIR-emitting fluorophore (these MI agents were named NIR-conPK and NIR-6T, respectively) [20], [21], [22]. For both these MI agents, we tested the following three criteria: Do they bind to TSPO in cell homogenates and intact cells? Does the signal that these MI agents emit fluctuate reliably as a function of changes in TSPO expression? How do fluctuations in the signal emitted by these MI agents correlate with a particular subtype of immune cell activation?

## Materials and Methods

### Chemicals

PK 11195 was from Sigma (St. Louis, MO), DAA1106 was synthesized as described [23], IFNγ, TNFα and TGFβ2 were from R&D systems (Minneapolis, MN), CellGro® was from Mediatech (Washington DC), DRAQ-5 from Axxora (San Diego, CA), WST1 from Roche Applied Science (Indianapolis, IN) and the IRDye 800CW dye was from LI-Cor biosciences (Lincoln, Nebraska).

### Synthesis and Characterization of MI Agent

Analogues of PK 11195 (conPK 11195: herein referred to as conPK) and DAA1106 (6-TSPOmbb732: herein referred to as 6T) were synthesized for conjugation to a fluorophore as described, and their chemical structure (Fig. S1), synthesis yield and characterization by spectroscopy have been previously reported [21], [24]. IRDye 800 CW is a fluorophore with absorption max = 778 nm and emission max = 806 nm. The dye bears a reactive N-hydroxy-succinimide reactive group allowing for facile conjugation to ligands of interest (Fig. S1). Briefly, a mixture of IRDye 800CW and conPK or 6-T was stirred in anhydrous dimethylsulfoxide in an Argon flushed vessel, in the dark at room temperature for 18 hrs. Reaction progress was followed by HPLC analysis using a C18 column (Varian, Inc. Palo Alto, CA) and eluting the compounds with acetonitrile: water. Compounds were further purified on an open alumina column eluting with acetonitrile: water, followed by an ion exchange column eluting with water. Pure fractions were then concentrated using rotary evaporation and dried with lyopholization. Nuclear Magnetic Resonance characterized the imaging agent, and Electrospray Ionization Mass Spectrometry calculated: 1364.01 (M^2+^ 681.95). Spectroscopic characterization in both water and DMSO consisted of both UV/vis Shimadzu 1701 spectrophotometer (Shimadzu Scientific Instruments, Columbia, MD) and fluorimetry (Photon Technology International, Birmingham, NJ).

### Cell Culture

DBT and BV-2 cells were grown in DMEM supplemented with FBS (10%). Mouse microglia in primary culture were prepared as described [25], [26] according to the guidelines of the Institutional Animal Care and Use committee of the University of Washington.

### qPCR

Total RNA from cells and tissues were extracted using Qiagen RNeasy. qPCR was performed using Taqman probes. Acidic ribosomal binding protein (ARBP) was used as a housekeeping gene [27] (control for equal RNA input) in all runs. Its levels did not change significantly between control and treatment conditions (data not shown). Thus, samples were normalized to the ARBP endogenous control using a Δct-based algorithm (ctTSPO-ctARBP) yielding arbitrary units that represent relative expression levels between samples. Primers were: ARBP: forward = 5′-GGTGTTTGACAACGACAGCATT-3′; reverse = 5′-CAGGGCCTGCTCTGTGATGT-3′. Mouse TSPO: forward = 5′-CAT GGG GTA TGG CTC CTA CA-3′; reverse = 5′-AGA CCC AAG GGA ACC ATA GC-3′

### Filtration Radioligand Binding Assay

Enriched membranes were prepared according to [28]. Briefly, mitochondrial-enriched membranes from DBT cells were prepared by lysing cells with ice-cold homogenization buffer (50 mM Tris, 1 mM EDTA and 3 mM MgCl_2_), followed by dounce homogenization (five gentle strokes). Homogenates were centrifuged (40,000×g, 15 min, 4°C) and cell pellet resuspended in 0.5 ml homogenization buffer, re-homogenized by dounce (five gentle strokes while on ice) and centrifuged again (40,000×g, 15 min, 4°C). Cell pellets were stored at −80°C until further use. Radioligand binding assays were performed in silanized glass test tubes (Kimble Glass Inc.). The following components were added: 50 µl of non-radiolabeled DAA1106 or PK 11195, 50 µl of [N-methyl-^3^H]-PK 11195 (PerkinElmer, Boston MA; 250 µCi, 84.8 Ci/mmol) and 100 µl of protein (50 µg, which started the incubation) for a total volume of 200 µl in binding buffer (50 mM Tris, 1 mM EDTA, 3 mM MgCl_2_ and 0.1% BSA), all of which was placed in a water bath (30°C) with mild agitation for 1 hr. Incubation was stopped by rapid filtration and rinsing with ice-cold binding buffer, using the Brandel harvester (Gaithersburg, MD) and Whatman GFB filter paper (Brandel, Gaithersburg MD). Filters were then transferred to 7 ml glass scintillation vials (VWR, West Chester PA). Five ml of Ecoscint XR scintillation fluid (National Diagnostics, Atlanta, GA) was added, followed by 10 sec vortexing and ∼18 hrs incubation at room temperature prior to determining the radioactivity with a scintillation counter (PerkinElmer, Boston MA).

### Intact Cell Binding Assay

We developed the intact cell binding assay described here for the first time: DBT or primary microglia cells at 5×10^4^ *per* 200 µl of DMEM+10% FBS were seeded in 96 well plates with optical bottom polymer (NUNC®). Twenty-four hours later, cells typically reached a density of ∼80% confluence. Cells were then rinsed once in MEM supplemented with 10% Cellgro®, pre-incubated with PK 11195, DAA1106 or vehicle, and then incubated with NIR-conPK or NIR-6T. Rinsing once with MEM/Cellgro stopped the incubation. Fluorescence at 800 nm was quantified with a Li-Cor Odyssey® Infrared Imaging System (LI-COR Biosciences, Lincoln, NE). Cells were then fixed in 4% PFA (10 min), rinsed twice with PBS/0.2% Triton (5 min) and four times with PBS. Cells were then incubated in Odyssey Blocking Buffer (90 min) and rinsed three times in PBS. DRAQ-5 (a dye that binds rapidly to DNA, is compatible with live cells and emits at 700 nM) was added for 5 min, allowing for precise determination of cell density in each well. These values were used to correct the signal read at 800 nM in each well. The values for non-specific binding were subtracted from total binding values (with data expressed in terms of relative fluorescence units, RFU).

### Cell Activation

DBT or primary microglia cells were treated with TNFα/IFNγ/TGFβ2 for 24 hrs, and supernatants (50 µl) collected for quantification of cytokine/chemokine levels using the mouse multiplex Beadlyte platform IS 100 instrument® (Upstate, Charlottesville, VA) and the Luminex® platform (Qiagen, Alameda, CA). For cell proliferation assay, we used WST-1 reagent. Specifically, 10 µl of WST-1 (*per* 100 µl of media) was added to each well and incubated for 2 hrs. Absorption was read with a Hewlett-Packard Spectracount® microplate reader (Palo Alto, CA) at 450 nm, using 590 nm as reference.

### Western Blots

Microglia cells treated with cytokines for 24 hrs were rinsed in PBS and lysed in Triton X 100 ice-cold lysis buffer (50 mM Tris HCl pH 7.5, containing 0.5% Triton X 100, 150 mM NaCl, 2 mM PMSF, one cocktail of protease inhibitors/50 ml, Roche Applied Science). The lysed cells were then centrifuged at 1300×g at 4C° (30 min). The resulting supernatant was then recovered for protein quantification and 40 µg of protein was sonicated and heated at 95C° prior to loading on to each lane of a NuPAGE Novex4–12%Bis-Tris gradient gel. After electrophoretically transferring the proteins to a nitrocellulose membrane, a Panceau S staining was conducted to visualize the proteins. The membrane was then blocked with 5% milk in PBS for one hr and immunoblotted for PBR (1∶1000, as kind gift from Dr. Vasilli Papadopolous) and GAPDH (glyceraldehyde-3-phosphate dehydrogenase, 1∶10000). The 18 Kd PBR and the 36 Kd GAPDH subunits were visualized using Pierce® Enhanced Chemiluminescent (ECL) Western Blotting Substrate (Thermo Scientific, Rockford, IL.) and horseradish peroxidase-goat anti-rabbit and horseradish peroxidase-goat anti-mouse secondary anti-sera used at 1∶10,000 and 1∶4,000 dilutions, respectively). The membrane was exposed over hyperfilm ECL (GE healthcare Amersham) and the densities of the bands quantified by Image J (Image Processing and Analysis in Java, NIH).

### Calculations and Statistical Analysis

Data are expressed as n = number of determinations (three *per* independent experiment). Statistical analysis, Kd and Ki values were calculated using non-linear regression with a one binding site hyperbola, where X is the concentration of the ligand, Y is specific binding and Bmax and Kd are determined using this equation: Y = B_max_ X/K_d_+X calculated by GraphPad Prism 4.

## Results

**Figure 1:**
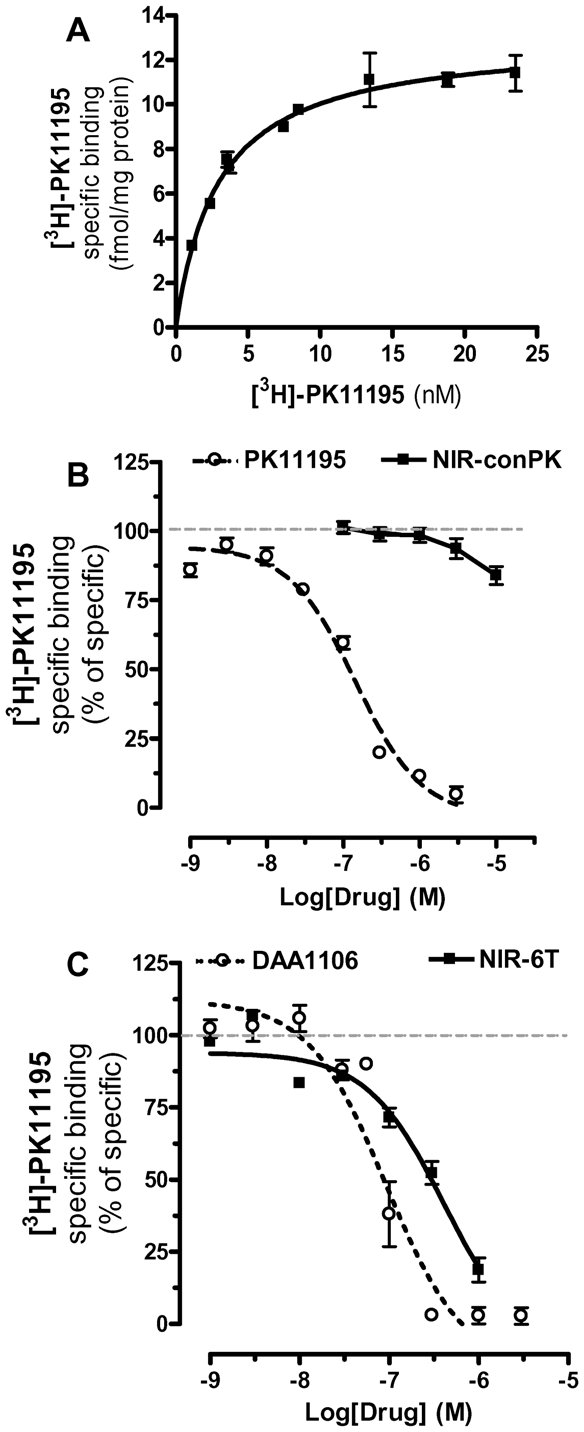
Ability of NIR-conPK and NIR-6T to compete for [^3^H]-PK 11195 binding to DBT cell homogenates. (**A**) Representative specific binding of [^3^H]-PK 11195 to DBT cell homogenates (average Bmax and Kd are in Table 2). (**B**) Competition of specific [^3^H]-PK 11195 binding to DBT cell homogenates by increasing concentrations of PK 11195 (dotted line) or NIR-conPK (solid line). Ki and EC_50_ values for PK 11195 competition were 41 and 93 nM respectively, and calculated using three independent experiments, each performed in triplicate. Ki value for NIR-conPK could not be reliably calculated, indicating that this MI agent does not bind to TSPO. (**C**) Competition of specific [^3^H]-PK 11195 binding to DBT cell homogenates by increasing concentrations of 6T (dotted line) and NIR-6T (solid line). Ki and EC_50_ values for 6T were 3.6 and 114 nM respectively and for NIR-6T 400 and 412 nM and were calculated using three independent experiments, each performed in triplicate.

To determine the pharmacological characteristics of the new MI agents targeting TSPO that we are studying, we required a cell line that expresses relatively high levels of TSPO. Using qPCR, we found that DBT cells, a highly malignant mouse astrocytoma cell line [29], express high levels of TSPO mRNA compared to two other mouse astrocytoma cell lines, D30 and D1A cells (Table 1) [30]. By comparison, levels of TSPO mRNA in DBT cells were also higher than in healthy mouse brain, mouse microglia in primary culture and BV-2 cells (a mouse microglia cell line) (Table 1) [31]. High TSPO expression in DBT cells was confirmed by filtration radioligand binding assays using [^3^H]-PK 11195 (we detected 14 pmol of TSPO *per* gram of protein, Fig. 1A, Table 2). Increasing concentrations of PK 11195 and DAA1106 competed for [^3^H]-PK 11195 binding with *Ki* of 2.0 and 0.2 nM, respectively (Fig. 1B, C), values that are well within the range of what has been reported [32]. These results show that DBT cells express relatively high levels of TSPO and therefore constitute a reliable cell model to determine the pharmacological characteristics of agents targeting TSPO with nanomolar affinity.

**Table 1:** qPCR determination of TSPO mRNA levels in cells and tissue.

Cell Line/Tissue Type	Δ CT	Fold Increase
DBT	1.93	21.4
Primary Microglia	3.58	6.8
D30	4.57	3.4
Spleen	4.81	2.9
D1A	5.17	2.3
BV-2	6.14	1.2
Brain	6.35	1

Cell and tissue mRNA were extracted, and TSPO and ARBP (house-keeping gene) mRNA levels determined by qPCR, as outlined in the material and methods section. Relative levels are expressed by normalized values using a Δct-based algorithm (ctTSPO-ctARBP). Fold increase compared to the lowest level of our sampling (brain) was calculated as outlined in the method section.

**Table 2:** Kd and Bmax of [^3^H]-PK 11195 binding using homogenates prepared from cytokine-treated DBT cells.

Conditions	Bmax	Ki
Control	14.0+/−1.1	4.3+/−0.65
IFNγ	17.0+/−2.6	4.7+/−0.7
TNFα	17.4+/−2.9	5.3+/−1.2
IFNγ+TNFα	17.6+/−2.4	5.2+/−0.9
TGFβ2	22.1+/−4.7	6.5+/−1.9

DBT cells incubated for 18–24 hrs with either vehicle (basal), TNFα (5 ng/ml), IFNγ (100 Ui/ml), TNFα+IFNγ, or TGFβ2 (1 ng/ml). Cells were harvested, homogenates prepared and [^3^H]-PK 11195 Kd and Bmax determined as outlined in the materials and methods section. Values are mean±SEM of 12–15 measurements (*i.e.* four or five independent experiments, each performed in duplicate). We found no significant difference in Kd and Bmax between basal and respective cytokine-treated conditions, as determined by ANOVA followed by Dunnett's.

Using DBT cell homogenates, we then tested whether NIR-conPK and NIR-6T compete for [^3^H]-PK 11195 binding, and found that NIR-conPK was relatively ineffective (with 10 µM displacing only ∼15% of [^3^H]-PK 11195 binding) (Fig. 1B), whereas NIR-6T competed for [^3^H]-PK 11195 binding with a *Ki* of 412 nM (Fig. 1C). These results show that the addition of an NIR moiety to con-PK greatly reduces its affinity at TSPO (to the point that it does not significantly bind to this protein anymore). Furthermore, the addition of an NIR moiety to 6T reduces its affinity by ∼2000 fold, but this MI agent still binds significantly to TSPO.

Radioligand binding performed on cell homogenate represents the gold standard for the pharmacological characterization of the binding of ligands to their targets [33].Yet evidence shows that the local microenvironments found in intact cells, which include local ionic concentrations and protein-protein interactions, greatly influences the pharmacological characteristics with which ligands bind to their targets [34], [35], [36]. This prompted us to test whether NIR-conPK and NIR-6T bind to TSPO in intact DBT cells. To do so, we developed an assay to measure specific binding in intact cells by quantifying NIR fluorescence, and varied the parameters required to optimize specific binding based on what had been described for radioligand binding assay performed in intact cells [37]. Specifically, we cultured DBT cells in 96 well plates, and optimized growing and total binding conditions by varying cell density, culture media, presence or absence of serum, type of plates, coatings plates with extracellular matrices, pre-incubation times and challenge *versus* competition experiments. We opted for plating the cells at ∼80% confluence at the time of experimentation, in defined media void of serum and in optical black-sided well plates, all of which resulted in the least non-specific binding to the plate, reduced light scattering and gave the greatest percent of specific binding. Thus, we incubated the cells under those conditions with either NIR-conPK or NIR-6T, rinsed the cells once (to remove unbound MI agents) and then quantified the amount of MI agent that had remained bound to intact cells with a LI-COR Odyssey® Infrared Imaging system [38].

**Figure 2:**
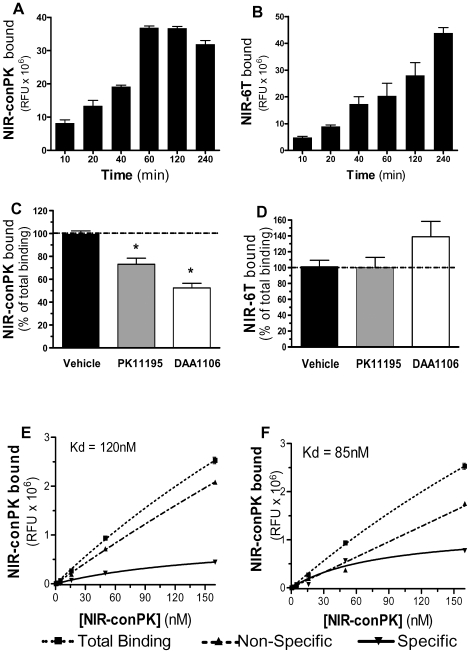
NIR-conPK and NIR-6T binding in intact DBT cells. (**A, B**) Kinetics of the total binding when labeling intact DBT cells with 100 nM of either NIR-conPK (A) or NIR-6T (B). (**C, D**) Ability of PK 11195 and 6T to compete for the binding of (C) NIR-conPK and (D) NIR-6T in intact DBT cells. DBT cells grown in 96 well plate were pre-incubated for 15 min with either vehicle (media with DMSO, *i.e.* total), PK 11195 (PK, 10 µM) or 6T (DAA, 10 µM), and then incubated with either (A) NIR-conPK (100 nM) or (B) NIR-6T (100 nM) for one hour. Cells were then rinsed and RFU at 800 nm read with an LI-COR Odyssey® Infrared Imaging system. Values are mean±SEM of nine measurements (*i.e.* three independent experiments, each performed in triplicate). *p<0.01, significantly different from total, as determined by ANOVA followed by Dunnett's. (**E, F**) Kd of NIR-conPK when using either (E) PK 11195 (10 µM) or (F) DAA1106 (10 µM) to determine non-specific binding. DBT cells grown in a 96 well plate were pre-incubated for 15 min with either media alone (*i.e.* total), or addition of PK 11195 or DAA1106, and then incubated with increasing concentration of NIR-conPK for one hour. Cells were then rinsed and RFU at 800 nm read with an LI-COR Odyssey® Infrared Imaging system. Values are mean±SEM of nine measurements (*i.e.* three independent experiments performed in triplicate). Solid line represents specific binding; dotted line with square shows total binding and dotted line with triangle shows non-specific binding.

The first parameter that we sought to determine was the amount of time required for these MI agents to reach binding equilibrium with intact cells. Total binding of NIR-conPK to DBT cells started to plateau after 60 min, while no such equilibrium was reached with NIR-6T, even after 4 hrs (Fig. 2As, B). Second, when using a 60 min incubation time, we verified that increasing concentration of both NIR-conPK and NIR-6T resulted in linear increases in fluorescence, and indeed found a linear correlation (Fig. S2). These results show that both MI agents bind to intact DBT cells in a time- and concentration-dependent manner, although NIR-6T does not reach equilibrium, even after several hrs.

To determine how much of this total binding of NIR-conPK and NIR-6T to intact cells is due to their specific binding to TSPO, we pre-treated intact DBT cells with either PK 11195 (10 µM) or DAA1106 (10 µM) to saturate TSPO, and assessed whether this pre-treatment reduces the amount of NIR-conPK and NIR-6T bound to intact cells. Remarkably, we found that the total binding of NIR-6T was not affected by either PK 11195 or DAA1106 (Fig. 2D), whereas the total binding of NIR-conPK was reduced by ∼25% by PK 11195 and ∼50% by DAA1106 (Fig. 2C), suggesting that NIR-conPK exhibits some amount of specific binding. Increasing the concentration of NIR-conPK yield a Kd of 120 nM when using an excess of PK 11195 (Fig. 2E) and a Kd of 85 nM when using an excess of DAA1106 (Fig. 2F). There are two possible interpretations of these results. One is that the pharmacological characteristics of NIR-conPK and NIR-6T binding to TSPO are opposite when using either cell homogenates or intact cells, with only NIR-6T binding to TSPO in cell homogenates, while only NIR-conPK binds to TSPO in intact cells. Another interpretation is that neither NIR-6T or NIR-conPK bind to TSPO in intact cells, and NIR-conPK binds to a protein distinct from TSPO that exhibits a pharmacological profile closely related to that of TSPO (since its binding is competed by both PK 11195 and DAA1106).

**Figure 3:**
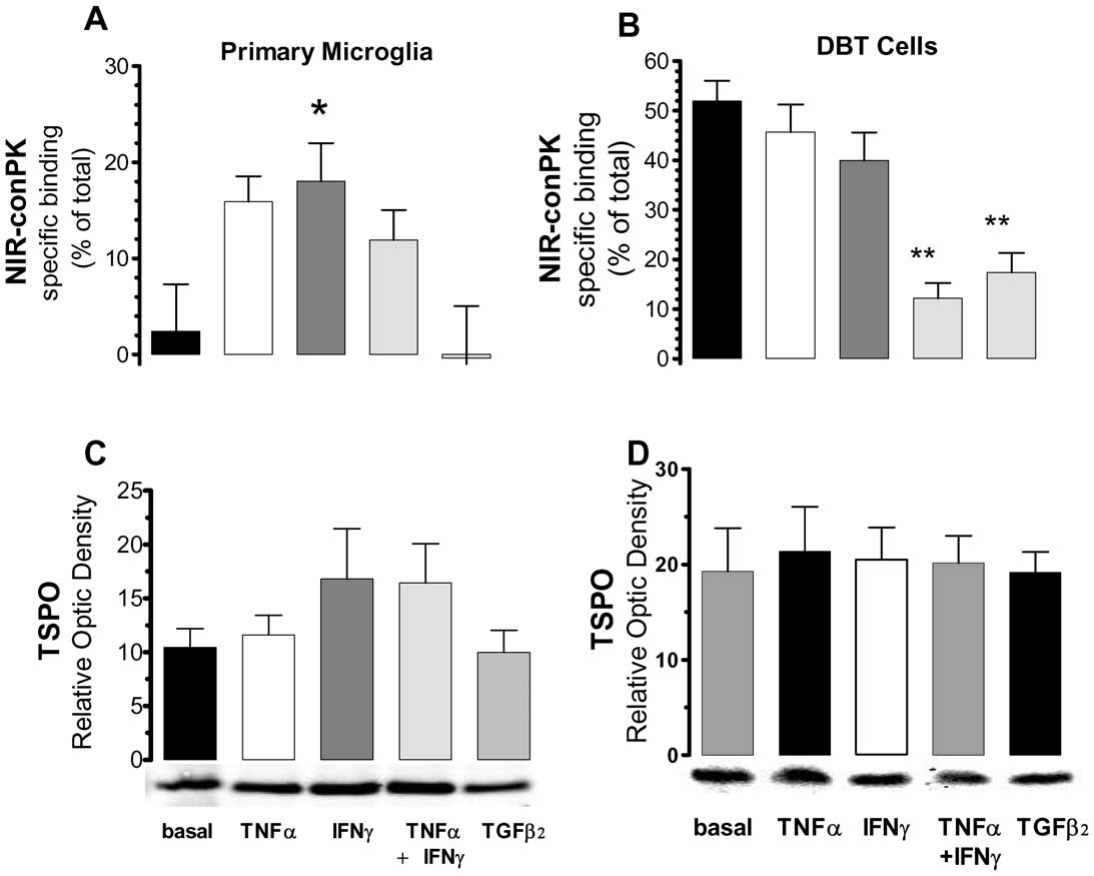
No correlation between changes in the specific binding of NIR-conPK and TSPO expression. (**A, C**) Primary mouse microglia and DBT (B, D) cells grown in 96 well plate were incubated for 18–24 hrs with either cell culture media (basal), TNFα (5 ng/ml), IFNγ (100 Ui/ml), TNFα plus IFNγ, or TGFβ2 (1 µg/ml). Cells then were pre-incubated for 15 min with either cell culture media (total binding) or DAA1106 (10 µM, non-specific binding), and then incubated with NIR-conPK for one hour. Cells were rinsed and RFU at 800 nm read with an LI-COR Odyssey® Infrared Imaging system. Specific binding was calculated by subtracting values of non-specific binding to total binding. Data are mean±SEM of 15 measurements (*i.e.* five independent experiments performed in triplicate). (**C, D**) Average optical density and representative western blot (below the graph) of TSPO expression in cytokine-treated primary microglia (C) or DBT (D) cells. Band corresponding to TSPO migrated at 18 kDa, as previously described [61].

To distinguish between these two possibilities, we sought to determine if the amount of specific binding measured for NIR-conPK correlates with TPSO expression. To perform these experiments, we used primary microglia in culture, because a positive correlation between immune cell activation and increases in TSPO expression has been reported [39], [40], [41], [42]. Thus we treated primary microglia in culture with TNFα, IFNγ or IFNγ/TNFα, all of which activate microglia toward a pro-inflammatory phenotype, or with TGFβ2, which inactivates microglia. Twenty four hrs later, we quantified the amount of specific binding obtained with NIR-conPK, as well as the expression levels of TSPO (by both qPCR and western blot). We found that IFNγ led to a significant increase in the amount of specific binding exhibited by NIR-conPK in intact microglia, whereas TNFα, TNFα+IFNγ and TGFβ2 did not (Fig. 3A). Importantly, the increase in NIR-conPK specific binding was not associated with a change in TSPO protein and mRNA (Fig. 3C and Fig. S3A). Please note that the treatment of microglia with these cytokines did not change cell density (Fig. S3C). Thus, changes in NIR-conPK specific binding do not correlate with changes in TSPO expression, and are likely due to the change in expression of a distinct protein.

We then performed similar experiments in DBT cells and also found no correlation, since here IFNγ+TNFα and TGFβ2 treatments led to a significant decrease in the amount of NIR-conPK specific binding in intact DBT cells (Fig. 3B), without concomitant change in TSPO expression or cell density (Fig. 3D and Fig. S3B,D). In this case, because of the ease of harvesting large amounts of DBT cells compared to primary microglial cells, we also performed radioligand binding experiments. Table 2 shows that cytokine treatment did not significantly change the amount of TSPO (Bmax), nor did it affect the affinity of PK 11195 at TSPO (Ki). This later point is important because such a change in affinity in response to cytokines has been reported and could have accounted for the increase in NIR-conPK specific binding following cytokine treatment [43] (see discussion). Together these results show that the cytokine-induced increases in NIR-conPK specific binding do not correlate with changes in either TSPO expression or the affinity of PK 11195 at TSPO, reinforcing the conclusion that this novel MI agent binds to a protein distinct from TSPO.

**Figure 4:**
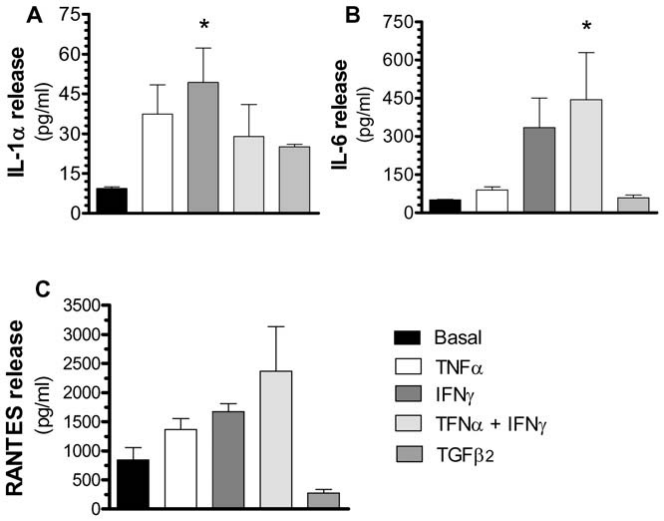
Cytokine and chemokine release by activated microglia. Primary mouse microglia were incubated for 18–24 hrs with either vehicle (basal), TNFα (5 ng/ml), IFNγ (100 Ui/ml), TNFα plus IFNγ, or TGFβ2 (1 ng/ml). Supernatant aliquots were recovered and amounts of (**A**) IL-1α, (**B**) IL-6 and (**C**) RANTES determined by Luminex beads. Values are mean±SEM of six measurements (*i.e.* three independent experiments, each performed in duplicate). **p<0.01; *p<0.05, significantly different from basal, as determined by ANOVA followed by Dunnett's.

In the final part of our study, we sought to better understand the neuroimmune function played by the activated microglia expressing more protein targeted by NIR-conPK. To do so, we sought to correlate increases in NIR-conPK specific binding with increases in immune effectors released by microglia. Specifically, under basal conditions, primary microglia in culture released 5 immune effectors (out of the 24 that we measured), namely IL-1α, IL-6, monocyte chemoattractant protein 1 (MCP-1), keratinocyte chemoattractant (KC) and RANTES (also referred to as chemokine (C-C motif) ligand 5, CCL5) (Fig. 4 and Table S1). When treating primary microglia with IFNγ, TNFα, IFNγ+TNFα or TGFβ2 for 24 hrs, we found that these stimuli lead to differential increases in the release of IL-1α, IL-6, and RANTES, and did not affect the release of KC and MCP-1 (Fig. 4 and Table S1). Remarkably, only the cytokine-induced release of IL-1α correlated with the cytokine-induced increase in NIR-conPK specific binding (Figs. 3A and 4A). These results suggest that only a particular subtype of microglia activation profile (here typified by IL-1α release and induced by IFNγ) is associated with an increase in the protein targeted by NIR-conPK.

## Discussion

Cancerous transformation and immune cell activation are associated with changes in the expression of hundreds to thousands of proteins [44], [45]. Some of these proteins, such as TSPO, constitute valuable targets for non-invasive *in vivo* MI and prognosis. However, when developing new MI agents, it is essential to systematically verify several criteria: that the selectivity and affinity of the MI agent at the targeted protein remains optimal, and that this MI agent reliably monitors any change in the expression of the targeted protein. Indeed, as we show in this study, the addition of an imaging moiety, such as a large hydrophobic fluorophore, to a ligand can greatly reduce its affinity toward the targeted protein, while increasing its affinity at a related protein. Thus, it is important to systematically verify that the amount of specific binding measured for a new MI agent indeed fluctuates concomitantly to changes in the expression of the targeted protein. Another challenge is to increase our understanding of the physiopathological significance of any changes in the expression of the targeted protein. Our aim was to address these questions for two MI agents, NIR-conPK and NIR-6T, originally developed to monitor TSPO expression in intact cells.

Radioligand binding (also known as membrane filtration receptor assay) constitutes the gold standard for determining the pharmacological characteristics of newly developed ligands [33], [37]. An obvious advantage of this approach is that it allows for the thorough manipulation and optimization of variables known to influence the amount of specific binding of ligands to their target, including: 1) adjusting the concentration of inorganic ions, 2) using chemically distinct labeled/unlabeled ligand for competition studies, 3) avoiding transport and agonist-induced receptor internalization mechanisms, and 4) excluding allosteric interactions with some endogenous binding partners [46]. We performed [^3^H]-PK 11195 binding studies with DBT cell homogenates and found pharmacological characteristics well within the range of what has been reported for this radioligand at TSPO (nanomolar Kd for [^3^H]-PK 11195, and nanomolar Ki for PK 11195 and DAA1106) [32]. Using this system, we show that NIR-6T competes for [^3^H]-PK 11195 binding at TSPO in cell homogenates; whereas NIR-conPK does not.

Conversely, MI binding performed on intact cells serves a different purpose, because it allows for the pharmacological characterization of a MI agent at its target under near-physiological conditions, whereby the ion concentrations and other parameters are set by the intact cells. Intact cell assays are advantageous with respect to studying receptors in an environment more closely related to the native one: the cell membrane has not been disturbed; intracellular components are in their intrinsic cytosolic context; and association with effector systems has not been perturbed. Disadvantages of this system include the complications linked to the transport of ligands into the cell, and the multiple regulatory mechanisms that receptors undergo during the course of a ligand reaching equilibrium (such as: internalization, down- or up-regulation, or changes in subcellular distribution). While G-protein coupled receptors have been somewhat characterized in respect to membrane *versus* intact cell binding, there is a scarcity of data reported on intracellular receptor binding in intact cells [47], [48], [49]. TSPO is a 5 transmembrane component of a pore complex located on the outer membrane of the mitochondria, associated with several other proteins [50]. While we found that NIR-6T binds with nanomolar affinity to TSPO in cell homogenates, it did not bind specifically to intact cells. We propose that this could be due to the relative bulkiness and hydrophilicity of the 800CW dye preventing the proper interaction of this MI agent to TSPO when assessed in its intact cell environment, for TSPO may be bound to regulatory proteins.

One of the most unexpected and interesting findings of our study is that NIR-conPK did not bind to TSPO in cell homogenates, but exhibited specific binding when applied to intact cells. Two sets of data suggest that the specific binding of NIR-conPK to intact cells is due to its interaction with a protein distinct, yet related to TSPO. First, the total binding of NIR-conPK in intact cells is competed-off by PK 11195 and DAA1106. Second, cytokine-induced changes in NIR-conPK specific binding does not correlate with cytokine-induced changes in TSPO expression. Further supporting our conclusion are studies reporting the existence of proteins pharmacologically-related to TSPO [51], [52], [53], one of which might represent the protein targeted by NIR-conPK that is described here. Thus, we propose that the addition of the 800CW dye to PK 11195 created a new interacting site, allowing for its nanomolar binding to a protein pharmacologically-related to TSPO.

The lack of systematic correlation between the cytokine-induced increase in NIR-conPK specific binding and TSPO expression could have been due to a cytokine-induced increase in the binding affinity of TSPO for PK 11195 and NIR-conPK. Indeed, such an example has been reported in human neutrophils, whereby N-formylmethionine-leucine-phenylalanine (f-MLP)-induces an increase in the affinity of TSPO for PK 11195 [43] (most likely through the induction of a TSPO binding partner [54], [55]). Furthermore, it should be emphasized that we used 100 nM of NIR-conPK in our MI binding experiments on intact cells and the Kd of NIR-conPK is 85 nM. Thus, a cytokine-induced increase in the affinity of TSPO for NIR-conPK would have been reflected by an increase in the amount of specific binding (100 nM lies within the linear portion of the dose-response). However, this possibility is unlikely because we found no change in the affinity of [^3^H]-PK 11195 at TSPO in cytokine-treated DBT cells (Table 2). Thus, the most parsimonious explanation remains that NIR-conPK targets a protein distinct from, yet related to TSPO.

While the protein targeted by NIR-conPK remains to be identified, our results show that this MI agent represents a valuable prototype because it can be used to monitor a particular subtype of microglial cell activation. The progression of most neuropathologies involves continuous changes in microglial cell phenotype, with each phenotype implementing a specific function executed by these macrophages during the development of complex neuroinflammatory responses [56]. For example, microglial cells adopt a pro-inflammatory profile when encountering IFNγ and TNFα, and are typically involved in the clearance of damaged cells; whereas microglia adopt an anti-inflammatory and repair phenotype when they encounter TGFβ2 [57]. We found that the pro-inflammatory cytokines IFNγ leads to an increase in both NIR-conPK specific binding and IL-1α release, a cytokine known to be involved in the early phases of many neuroinflammatory responses [58], [59], [60]. Thus, fluctuations in NIR-conPK signal could be used to monitor the early phases of neuroinflammatory responses. However, before achieving this goal, the affinity of NIR-conPK at its target will have to be improved as to enhance its *in vivo* imaging properties. The development of an improved NIR-conPK that allows for the monitoring of early neuroinflammatory responses would represent a valuable asset for the diagnostic of many neuropathologies.

In conclusion, our results show that NIR-6T does not represent a viable MI agent to monitor TSPO expression in intact cells, and that NIR-conPK most likely binds to a protein distinct from, yet related to TSPO. Our results also suggest that NIR-conPK represents a valuable prototype for the development of future MI agents aimed at monitoring changes in a particular subtype of microglial cell activation process. As such, increasing the number of MI agents monitoring the various phases that microglial cell activation typically undergo during neuroinflammatory responses will be useful for monitoring the progression of various neuropathologies, and assessing the efficacy of their treatments.

## Supporting Information

Figure S1 Chemical structure of PK 11195 and DAA1106 analogues. (A) conPK is a conjugable analogue of PK 11195. In conPK, the N-methyl, N-isobutyl amide is replaced with with hexane diamine for conjugation to imaging agents. Arrows indicate methyl groups on PK 11195 that are not present on conPK. (B) 6-TSPOmbb732 (6-T) is a conjugable analog of DAA1106. One methoxy group in DAA1106 is replaced with hexane diamine for easy conjugation to signaling moieties.
(0.80 MB TIF)

Figure S2 Linearity of dye fluorescence. Increasing concentration of NIR-conPK (A) and NIR-6T (B) in black sided optical 96 well plates (in triplicate) was read on the Odyssey Imaging platform. Relative fluorescence values revealed a linear relationship between dye concentration and emitted fluorescence.
(0.78 MB TIF)

Figure S3 TSPO expression by qPCR and cell density. (A) Primary mouse microglia and (B) DBT cells were incubated for 18–24 hrs with either vehicle (basal), TNFα (5 ng/ml), IFNγ (100 Ui/ml), TNFα plus IFNγ, or TGFβ2 (1 ng/ml), and TSPO mRNA levels quantified by qPCR, as described in Table 1 legend. Cell density of cytokine-treated (C) primary mouse microglia and (D) DBT cells was determined using WST-1, as described in the Materials and Methods section.
(0.84 MB TIF)

Table S1 Chemokine release by activated microglia. Primary mouse microglia were incubated for 18–24 hrs with either vehicle (basal), TNFα (5 ng/ml), IFNγ (100 Ui/ml), TNFα plus IFNγ, or TGFβ2 (1 µg/ml). Supernatant aliquots were recovered and amount of KC and MCP-1 (pg/ml) determined by Luminex beads. Values are mean±SEM of six measurements (i.e. three independent experiments, each performed in duplicate). We found no significant differences in the release of these chemokines between basal and respective cytokine-treated conditions, as determined by ANOVA followed by Dunnett's.
(0.86 MB TIF)
