# Supplementary figures and images for: Binding of NIR-conPK and NIR-6T to Astrocytomas and Microglial Cells: Evidence for a Protein Related to TSPO
Source: PLoS One. 2009 Dec 18;4(12):e8271. doi: 10.1371/journal.pone.0008271 (PMC2792720; doi:10.1371/journal.pone.0008271)

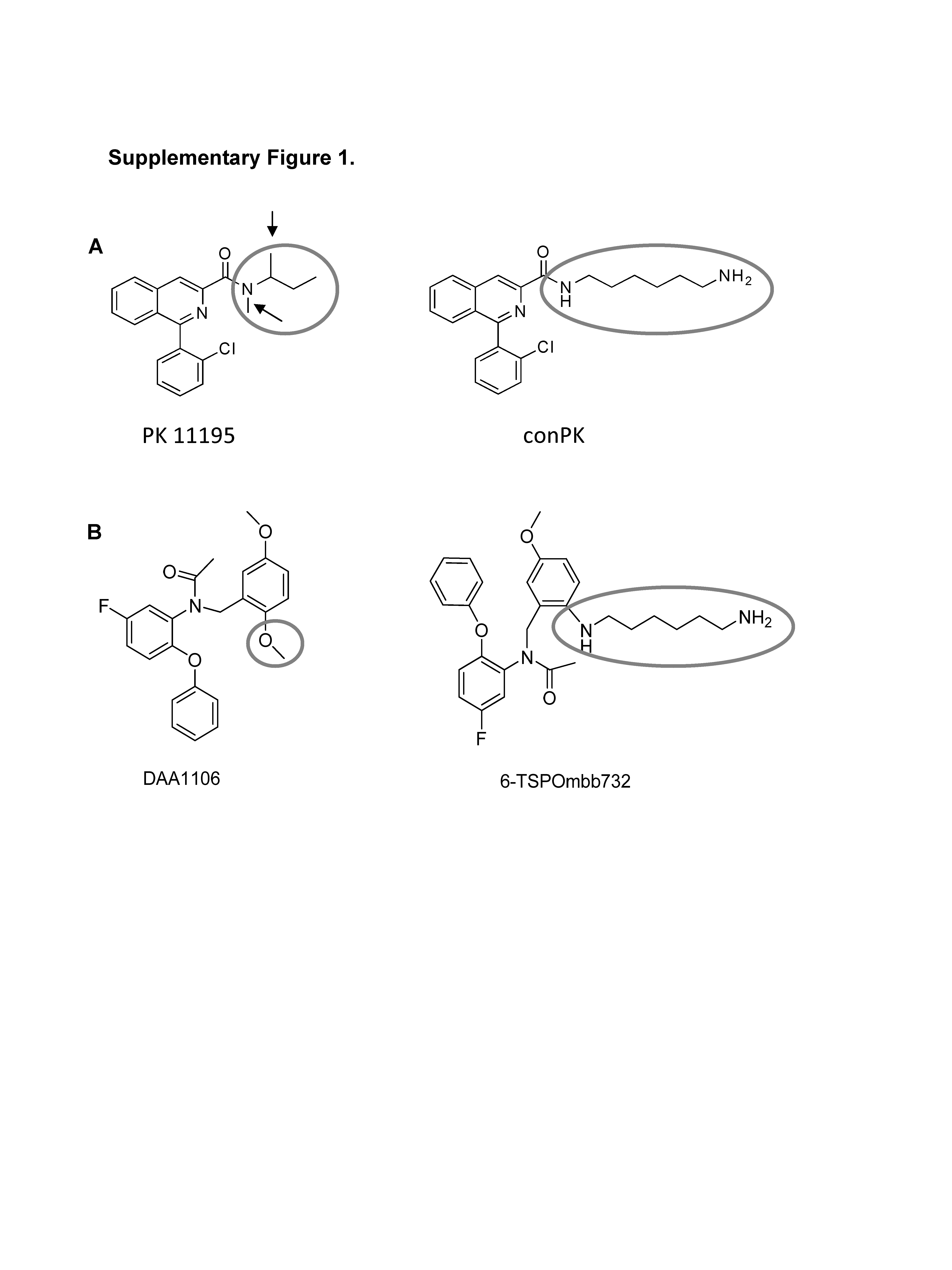

Supplement: Figure S1 — Chemical structure of PK 11195 and DAA1106 analogues. (A) conPK is a conjugable analogue of PK 11195. In conPK, the N-methyl, N-isobutyl amide is replaced with with hexane diamine for conjugation to imaging agents. Arrows indicate methyl groups on PK 11195 that are not present on conPK. (B) 6-TSPOmbb732 (6-T) is a conjugable analog of DAA1106. One methoxy group in DAA1106 is replaced with hexane diamine for easy conjugation to signaling moieties. (0.80 MB TIF) [file pone.0008271.s001.tif]

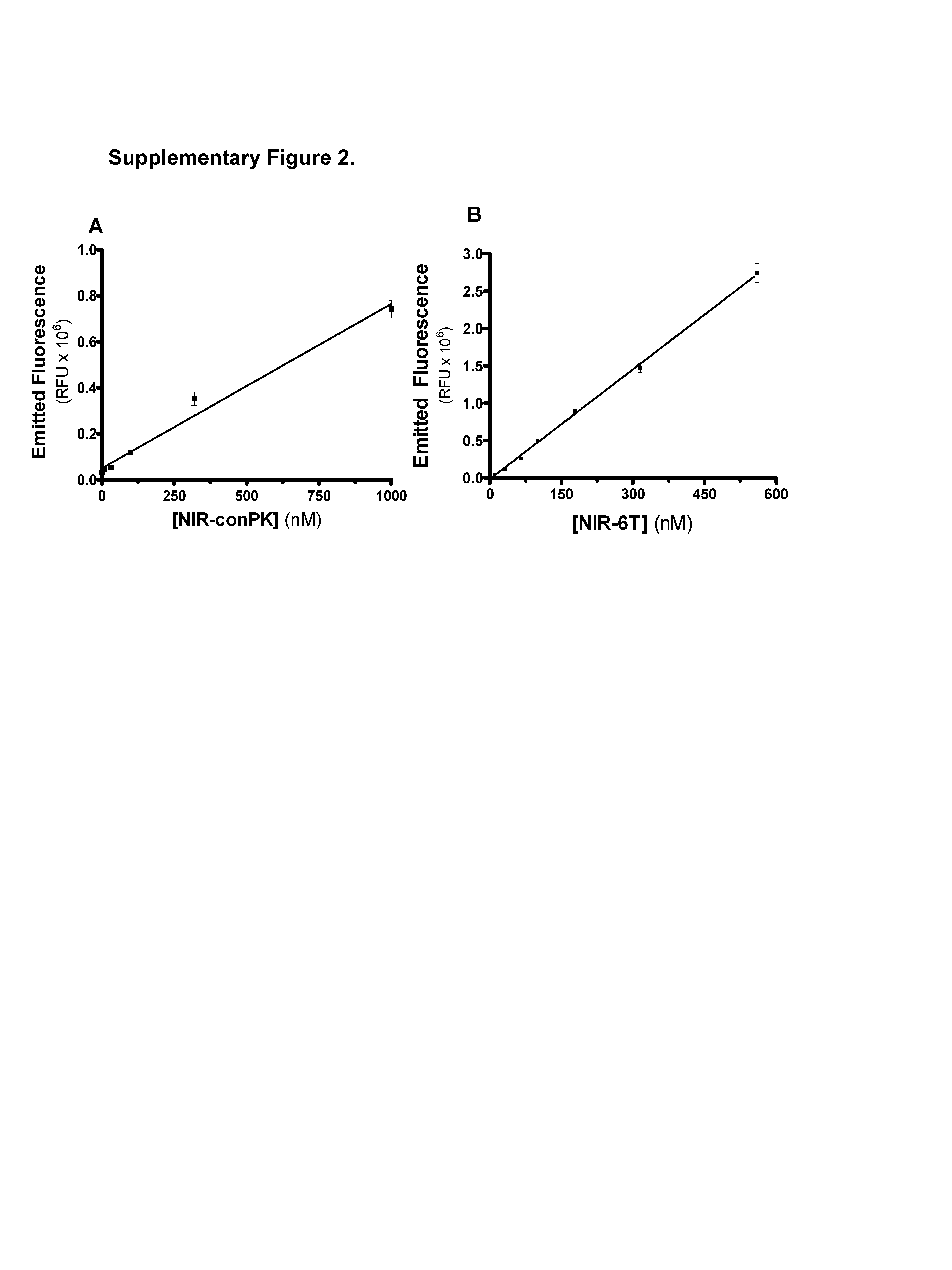

Supplement: Figure S2 — Linearity of dye fluorescence. Increasing concentration of NIR-conPK (A) and NIR-6T (B) in black sided optical 96 well plates (in triplicate) was read on the Odyssey Imaging platform. Relative fluorescence values revealed a linear relationship between dye concentration and emitted fluorescence. (0.78 MB TIF) [file pone.0008271.s002.tif]

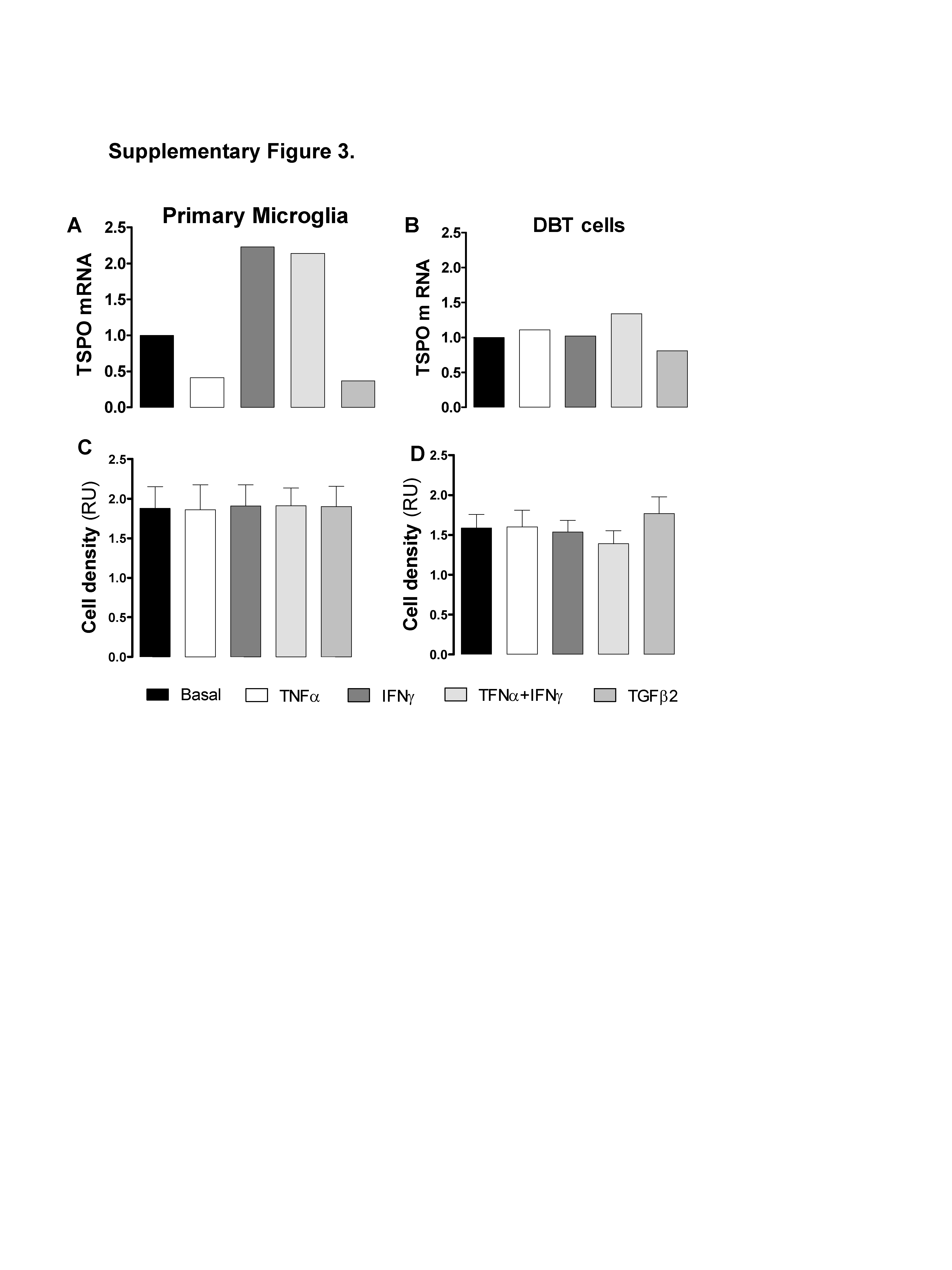

Supplement: Figure S3 — TSPO expression by qPCR and cell density. (A) Primary mouse microglia and (B) DBT cells were incubated for 18–24 hrs with either vehicle (basal), TNFα (5 ng/ml), IFNγ (100 Ui/ml), TNFα plus IFNγ, or TGFβ2 (1 ng/ml), and TSPO mRNA levels quantified by qPCR, as described in Table 1 legend. Cell density of cytokine-treated (C) primary mouse microglia and (D) DBT cells was determined using WST-1, as described in the Materials and Methods section. (0.84 MB TIF) [file pone.0008271.s003.tif]

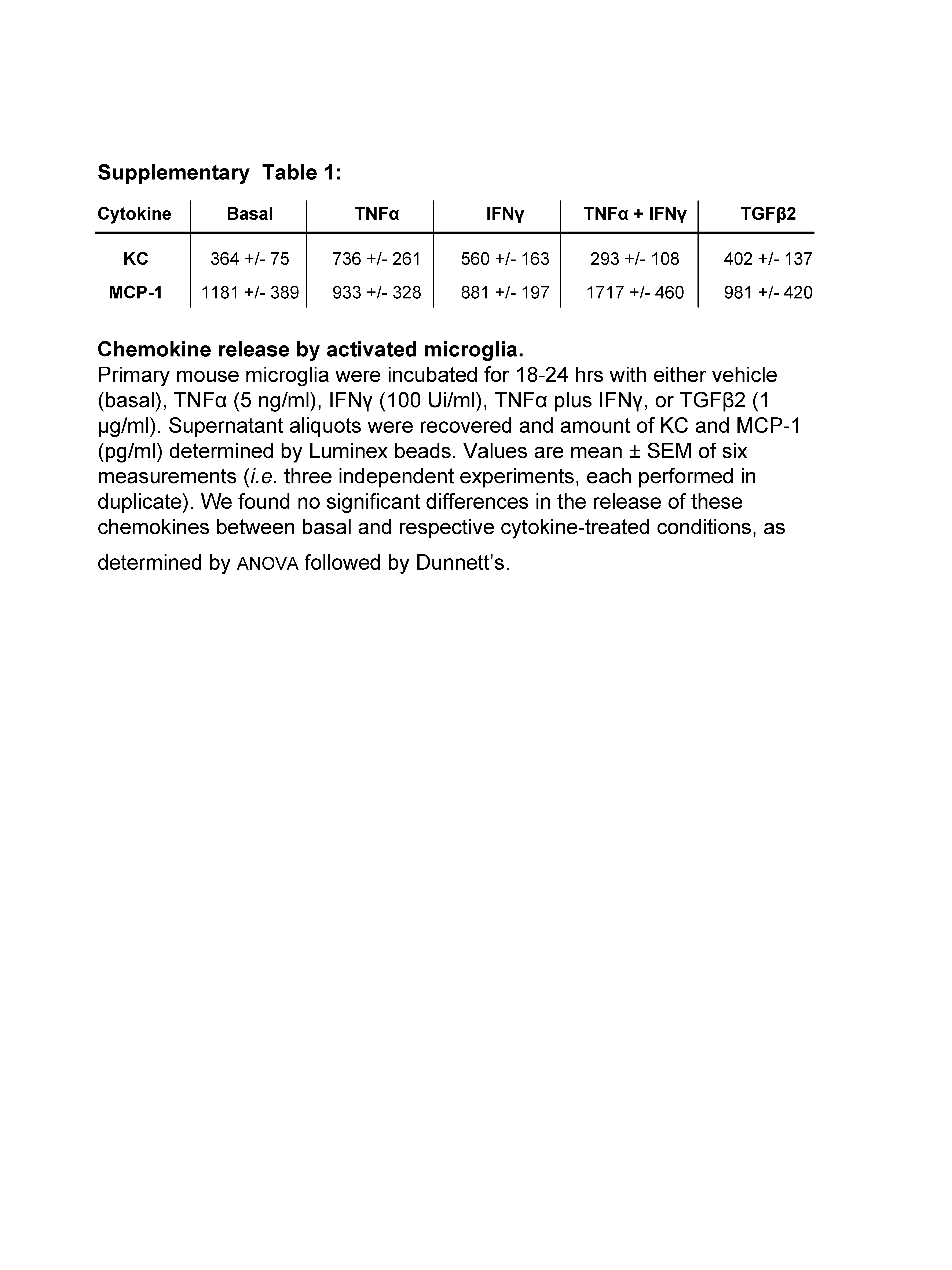

Supplement: Table S1 — Chemokine release by activated microglia. Primary mouse microglia were incubated for 18–24 hrs with either vehicle (basal), TNFα (5 ng/ml), IFNγ (100 Ui/ml), TNFα plus IFNγ, or TGFβ2 (1 µg/ml). Supernatant aliquots were recovered and amount of KC and MCP-1 (pg/ml) determined by Luminex beads. Values are mean±SEM of six measurements (i.e. three independent experiments, each performed in duplicate). We found no significant differences in the release of these chemokines between basal and respective cytokine-treated conditions, as determined by ANOVA followed by Dunnett's. (0.86 MB TIF) [file pone.0008271.s004.tif]
